# Leukocyte mitochondrial DNA copy number, anthropometric indices, and weight change in US women

**DOI:** 10.18632/oncotarget.10325

**Published:** 2016-06-29

**Authors:** Shasha Meng, Shaowei Wu, Liming Liang, Geyu Liang, Edward Giovannucci, Immaculata De Vivo, Hongmei Nan

**Affiliations:** ^1^ Department of Epidemiology, Harvard T. H. Chan School of Public Health, Boston, MA, USA; ^2^ Department of Environmental Health, Harvard T. H. Chan School of Public Health, Boston, MA, USA; ^3^ Channing Division of Network Medicine, Brigham and Women's Hospital and Harvard Medical School, Boston, MA, USA; ^4^ Key Laboratory of Environmental Medicine Engineering, Ministry of Education, School of Public Health, Southeast University, Nanjing, Jiangsu, China; ^5^ Department of Nutrition, Harvard T. H. Chan School of Public Health, Boston, MA, USA; ^6^ Department of Epidemiology, Richard M. Fairbanks School of Public Health, Indiana University, Indianapolis, IN, USA; ^7^ Indiana University Melvin and Bren Simon Cancer Center, Indianapolis, IN, USA

**Keywords:** mitochondrial DNA copy number, anthropometric indices, weight change, telemere length

## Abstract

**Objectives:**

To examine the association between leukocyte mitochondrial DNA copy number (mtCN) and different anthropometric indices as well as weight changes; and to compare mtCN and telomere length with respect to their associations with BMI and age.

**Design:**

Population based cohort study.

**Setting:**

Nurses' Health Study, an ongoing prospective cohort study of 121,700 nurses enrolled in 1976; in 1989-1990 a subset of 32,826 women provided blood samples.

**Participants:**

1,700 disease-free US women from case-control studies nested within the Nurses' Health Study with mtCN and telomere length measured who also have anthropometric measurements.

**Main outcome measure:**

Relative mtCN and telomere lengths in peripheral blood leukocytes measured by quantitative real time polymerase chain reaction and various anthropometric measurements data from initial questionnaire.

**Results:**

Leukocyte mtCN was inversely associated with current weight (LS means Q1-Q4: 0.07, 0.04, 0.03, −0.17; *P trend* =0.002), waist size (LS means Q1-Q4: 0.06, 0.05, −0.04, −0.06; *P trend* = 0.04), BMI (LS means normal light, normal heavy, overweight, pre-obese, obese: 0.11, −0.01, −0.04, 0.04, −0.25; *P _trend_*<0.0001), and waist-hip ratio (WHR) (LS means Q1-Q4: 0.06, 0.08, −0.04, −0.06; *P trend* = 0.03). A one-unit decrease in mtCN z score was equivalent to approximately 3.5 pounds of weight gain for an adult of 5′10″. In addition, weight gain was bi-directionally and inversely associated with mtCN. Moreover, mtCN was strongly positively correlated with telomere length (LS means Q1-Q4: −0.02, 0.09, 0.11, 0.33; *P trend* <0.0001). MtCN was inversely associated with BMI even after adjusting for telomere length (*P trend* =0.003), while telomere length was not associated with BMI. On the other hand, telomere length was inversely associated with age after adjusting for mtCN (*P trend* =0.04), while mtCN was not associated with age.

**Conclusions:**

Our results provide compelling evidence for a potential bi-directional temporal relationship between mitochondrial-mediated oxidative stress-defense mechanisms and weight change.

## INTRODUCTION

Mitochondria are organelles in the cytoplasm within a cell whose main functions include energy metabolism, free-radical production, calcium homeostasis, and apoptosis [1]. Mitochondria are also the primary site for the degradation of fatty acids, through β-oxidation [2]. Mitochondrial DNA (mtDNA) located close to the source of reactive oxidative stress (ROS) production is extremely susceptible to oxidative damage due to its lack of either protective histones or introns and the scarcity of efficient DNA repair mechanisms [3, 4]. Associations between leukocyte mtDNA copy number (mtCN) and various health outcomes have been demonstrated in multiple prospective studies, where lower mtCN was significantly associated with poorer cognitive abilities, physical strength, and self-rated health as well as higher prevalent frailty and all-cause mortality [5–7]. MtCN has also been suggested as a contributor to many cancer types [8]. This body of evidence suggests that mtCN in leukocytes may serve as a candidate biomarker for oxidative stress-related general health outcomes.

Obesity, defined as a body mass index (BMI) of 30kg/m^2^ or higher, is a major risk factor in the pathogenesis of many chronic diseases such as cardiovascular diseases, diabetes, and some cancers (e.g., endometrial, breast, and colon) [9]. An important pathway through which obesity contributes to these conditions is by increasing systemic inflammation and oxidative stress [10]. Adipose tissue is a main source of cytokines and adipokines, which increase systemic oxidative stress. It has been suggested that the activation of the innate immune system in adipose tissue can also trigger a systemic acute-phase inflammation response, which can be further promoted by free radicals generated by activated immune cells [11, 12]. A previous study among insulin-resistant subjects showed that markers of mitochondrial biogenesis and metabolism are lower in those who are overweight and obese [13]. Furthermore, a study of 94 healthy young Korean individuals identified an independent association between mtDNA content and visceral adiposity [14].

Telomere length is a key marker of cellular and biologic aging [15, 16]. Though both mtCN and telomere length can reflect the cumulative burden of oxidative stress and inflammation, it remains unclear how they differ in their sensitivities to oxidative stress. A biological connection between telomere damage and mitochondrial dysfunction was recently discovered in mouse models; it has been shown that inhibition of telomere shortening-induced activators (e.g., PGC1α and PGC1β) may consequently impair mitochondrial biogenesis and function [17–19]. Nevertheless, population-based studies investigating the interaction of mtCN and telomere length are lacking. Given the available evidence of associations between telomere length and anthropometric measurements [20–22] and the simultaneous availability of mtCN and telomere length measurements in our dataset, we prospectively assessed the associations among leukocyte mtCN, telomere length, and commonly used anthropometric measures as well as weight changes in 1,700 healthy women from the Nurses' Health Study (NHS). Furthermore, we also compared the sensitivity of mtCN and telomere length in relation to age and obesity.

## RESULTS

The descriptive characteristics of the 1,700 women in our population according to the mtCN quartiles are provided in Table 1. In general, mtCN z score quartiles were similar with respect to age, postmenopausal status, postmenopausal hormone use, oral contraceptive use, parity, total activity/week, alcohol consumption, and total calorie intake. A higher percentage of current smokers was found in the lower mtCN quartiles.

**Table 1:** Basic characteristics of the study population at blood collection^a^

	Quartiles of mtCN z score			
	Quartile 1 (n=426)	Quartile 2 (n=427)	Quartile 3 (n=427)	Quartile 4 (n=426)
Age at blood draw, mean (SD)	58.03 (6.91)	57.96 (6.56)	57.43 (6.99)	57.75 (6.71)
Postmenopausal status, n (%)	335 (78.63)	345 (80.90)	332 (77.74)	331 (77.61)
Age at menopause	49.96 (8.82)	50.65 (10.05)	51.22 (9.66)	50.53 (7.90)
Postmenopausal hormone use				
Never, n (%)	175 (41.37)	188 (44.34)	207 (49.40)	190 (45.82)
Past, n (%)	71 (16.78)	65 (15.33)	62 (14.80)	71 (16.95)
Current, n (%)	177 (41.84)	171 (40.33)	150 (35.80)	158 (37.71)
Oral contraceptive use, n (%)	196 (45.93)	195 (45.62)	200 (46.87)	190 (44.56)
Parity, n (%)	27 (6.26)	21 (4.87)	26 (6.11)	35 (8.31)
Smoking status				
Never, n (%)	171 (40.24)	171 (40.05)	191 (44.84)	171 (40.24)
Past, n (%)	154 (36.24)	171 (40.05)	168 (39.44)	178 (41.88)
Current, n (%)	100 (23.53)	85 (19.91)	67 (15.73)	76 (17.88)
Physical activity (MET hours/week)^b^, mean (SD)	15.08 (17.08)	12.87 (15.23)	15.14 (18.12)	15.41 (18.07)
Alcohol intake, gm/day, mean (SD)	6.09 (10.14)	5.16 (8.90)	4.79 (8.23)	6.11 (11.06)
Total Calories, kcal/day, mean (SD)	1749.42 (468.66)	1746.08 (460.26)	1783.67 (501.00)	1728.59 (468.80)

a All variables are measured at blood draw and are standardized to the age distribution of the study population.

b MET denotes metabolic equivalent. Met-hours = sum of the average time/week in each activity x MET value of each activity. One MET, the energy spent sitting quietly, is equal to 3.5 ml of oxygen uptake per kilograms of body weight per minute for a 70-kg adult.

We calculated the Spearman correlation coefficients (*r_s_*) between mtCN z score and anthropometric variables as well as other demographic and lifestyle factors (Table 2). There were significant inverse associations between mtCN and weight (*r_s_*= -0.09, *P*=0.0002), waist measurement (*r_s_*= -0.06, *P*=0.01), BMI (*r_s_*= -0.10, *P*<0.0001), and waist-to-hip ratio (*r_s_*= -0.05, *P*=0.03). In addition, mtCN was inversely correlated with pack years of smoking (*r_s_*= -0.07, *P*=0.03) among current smokers and positively correlated with telomere length (*r_s_*=0.12, *P*=0.0001).

**Table 2:** Age-adjusted Spearman correlations between relative mitochondrial DNA copy number (z score) and various characteristics at blood collection

Characteristic (n=1,700)	*r*_s_^a^	*p*-value
Weight, kg^b^	−0.09	0.0002
Height at baseline, inches	−0.03	0.27
Waist measurement, inches	−0.06	0.01
Hip measurement, inches	−0.04	0.10
BMI, kg/m^2^	−0.10	<.0001
Waist to hip ratio	−0.05	0.03
Waist to height ratio	−0.05	0.05
Age	−0.02	0.48
Pack years of smoking^c^	−0.07	0.03
Physical activity, MET hours/week	0.02	0.33
Total calories, kcal/day	−0.02	0.42
Alcohol intake, gm/day	0.01	0.70
Relative telomere length	0.12	0.0001

a Spearman correlation coefficient, adjusted for age at blood collection

b Adjusted for age at blood collection and height

c Among current smokers

The associations of mtCN with common anthropometric variables categories including weight, waist circumference, BMI, and waist-to-hip ratio are presented in Table 3. Inverse associations were observed between mtCN and categories of weight (LS means Q1-Q4: 0.07, 0.04, 0.03, −0.17; *P_trend_*=0.002), waist circumference (LS means Q1-Q4: 0.06, 0.05, −0.04, −0.06; *P_trend_*=0.04), BMI (LS means normal light, normal heavy, overweight, pre-obese, obese: 0.11, −0.01, −0.04, 0.04, −0.25; *P_trend_*<0.0001), and waist-to-hip ratio (LS means Q1-Q4: 0.06, 0.08, −0.04, −0.06; *P_trend_*=0.03). We excluded the underweight group because of potential differences in their etiology. In addition, linear regression indicated that a one-unit decrease in mtCN z score was equivalent to approximately 3.5 pounds of weight gain for an adult of 5′10″ (*P*<0.0001).

**Table 3:** Associations of mtCN z score with anthropometric measurements and telomere length

Anthropometric variables	Relative mtCN z score			
	N	Mean (95% CI)	*P*-value	*P* for trend
Weight (kg)^a^				
Q1 (<59.5)	433	0.07 (−0.06, 0.20)	Ref	0.002
Q2 (59.5-65.5)	375	0.04 (−0.09, 0.17)	0.64	
Q3 (65.5-74.5)	452	0.03 (−0.10, 0.16)	0.52	
Q4 (74.5>)	428	−0.17 (−0.30, −0.04)	0.0008	
Waist (inch)^a^				
Q1 (<28)	357	0.06 (−0.07, 0.20)	Ref	0.04
Q2 (29-31)	349	0.05 (−0.08, 0.19)	0.90	
Q3 (31-32)	600	−0.04 (−0.16, 0.08)	0.11	
Q4 (>33)	382	−0.06 (−0.19, 0.08)	0.10	
BMI^b^				
Normal light (18.5-23)	527	0.11 (−0.01, 0.23)	Ref	<0.0001
Normal heavy (23-25)	366	−0.01 (−0.15, 0.13)	0.07	
Overweight (25-27.5)	337	−0.04 (−0.18, 0.09)	0.02	
Pre-obese (27.5-30)	193	0.04 (−0.13-0.20)	0.36	
Obese (>30)	240	−0.25 (−0.41, −0.10)	<0.0001	
Waist-hip ratio^b^				
Q1 (<0.735)	436	0.06 (−0.07, 0.19)	Ref	0.03
Q2 (0.737-0.775)	249	0.08 (−0.07, 0.24)	0.73	
Q3 (0.778-0.822)	581	−0.04 (−0.16, 0.08)	0.12	
Q4 (>0.824)	422	−0.06 (−0.19, 0.07)	0.09	
Telomere^b^				
Q1 (<0.696)	250	−0.02 (−0.18, 0.14)	Ref	<0.0001
Q2 (−0.689-0.026)	256	0.09 (−0.07, 0.25)	0.19	
Q3 (−0.020-0.701)	254	0.11 (−0.05, 0.28)	0.11	
Q4 (>0.707)	251	0.33 (0.17, 0.50)	<0.0001	

a Generalized linear models are adjusted for age at blood draw, height, smoking, menopausal status, PMH use, and alcohol consumption.

b Generalized linear models are adjusted for age at blood draw, smoking, menopausal status, PMH use, and alcohol consumption.

We further examined the bi-directional associations between mtCN and weight changes with different lag time (5 years and 10 years). As shown in Table 4, women with more than 20kg of weight gain since age 18, more than 10kg of weight gain since baseline (1976 to 1989), more than 10kg of weight gain 10 years prior to blood collection (1979-1989), and more than 5kg of weight gain 5 years prior to blood collection (1984-1989) had significantly lower levels of mtCN compared to women with stable weight, defined as ±2kg of weight change (weight change since age 18, LS means from weight maintenance to weight gain>20kg=0.07, 0.02, −0.13; *P_trend_*=0.02; weight change since baseline, LS means from weight maintenance to weight gain>10kg=0.05, 0.01, −0.16; *P_trend_*=0.01; weight change since 10 years prior to the blood collection, LS means from weight maintenance to weight gain >10kg=0.03, 0.03, −0.21; *P_trend_*=0.03; weight change since 5 years prior to the blood collection, LS means from weight maintenance to weight gain>5kg=0.06, −0.02, −0.11; *P_trend_*=0.01). Lower mtCN at blood collection was also found to be inversely associated with future weight gain: women with lower mtCN gained more weight in the next 5 years and 10 years after blood collection (weight gain in the next 5 years, mtCN Q1-Q4 weight gain in pounds =4.13, 4.05, 3.42, 3.23; *P_trend_*=0.003; weight gain in the next 10 years, mtCN Q1-Q4 weight gain in pounds=5.63, 5.24, 4.91, 4.45; *P_trend_*=0.01). We excluded the weight-loss members from the trend test, because weight loss may be due to a variety of reasons and take place via other physiological pathways besides weight gain.

**Table 4:** Associations between mtCN z score and weight changes^a^

Weight change	n	Relative mtCN z score or weight change in pounds	*P* for trend
		Mean (95% CIs)	
Weight change since 18			
Weight loss	155	0.04 (−0.14, 0.21)	
Weight maintenance	134	0.07 (−0.12, 0.26)	0.02
Weight gain between 2-20kg	1028	0.02 (−0.09, 0.13)	
Weight gain >20kg	292	−0.13 (−0.28, 0.01)	
Weight change since baseline (1976 to 1989)			
Weight loss	186	0.05 (−0.12, 0.23)	
Weight maintenance	362	0.05 (−0.08, 0.19)	0.01
Weight gain between 2-10kg	816	0.01 (−0.10, 0.12)	
Weight gain >10kg	307	−0.16 (−0.30, −0.01)	
Weight change from 1979-1989			
Weight loss	257	0.01 (−0.15, 0.16)	
Weight maintenance	420	0.03 (−0.10, 0.17)	0.03
Weight gain between 2-10kg	764	0.03 (−0.08, 0.15)	
Weight gain >10kg	173	−0.21 (−0.38, −0.04)	
Weight change from 1984 to 1989			
Weight loss	279	−0.001 (−0.15, 0.15)	
Weight maintenance	531	0.06 (−0.06, 0.19)	0.01
Weight gain between 2-5kg	423	−0.02 (−0.15, 0.11)	
Weight gain >5kg	317	−0.11 (−0.25, 0.03)	
Weight change from 1989 to 1994			
mtCN Q1	326	4.13 (3.45, 4.80)	0.003
mtCN Q2	326	4.05 (3.37, 4.74)	
mtCN Q3	356	3.42 (2.75, 4.09)	
mtCN Q4	333	3.23 (2.55, 3.91)	
Weight change from 1989-1999			
mtCN Q1	287	5.63 (4.75, 6.50)	0.01
mtCN Q2	288	5.24 (4.35, 6.13)	
mtCN Q3	313	4.91 (4.04, 5.78)	
mtCN Q4	295	4.45 (3.57, 5.32)	

a Linear regression models are adjusted for age at blood draw, smoking, menopausal status, PMH use, and alcohol consumption. Blood collection year: 1989.

Because telomere length was found to be associated with BMI in previous studies of the NHS [21, 22], we examined the association between mtCN and telomere length, and found a strong positive association as presented in Table 3 (mtCN LS means across Q1-Q4 of telomere length: −0.02, 0.09, 0.11, 0.33; *P_trend_* <0.0001). In Table 5, we showed a comparison of mtCN and telomere length with respect to their associations with BMI and age in the year of blood collection. In our data, mtCN was associated with BMI even after adjusting for telomere length (LS means normal, overweight, and obese: 0.18, 0.10, −0.09; *P_trend_*=0.003), while telomere length was not associated with BMI. On the other hand, telomere length was inversely associated with age after adjusting for mtCN (LS means <50, 50-55, 55-60, 60-65, >65: 0.22, −0.02, −0.07, −0.09, −0.15; *P_trend_*=0.04), while mtCN was not associated with age.

**Table 5:** Comparative analyses between mtCN and telomere length in relation to age and anthropometric variables

Anthropometric variables	N	MtCN z score		MtCN z score^a^		Telomere z score		Telomere z score^b^	
		Mean (95% CI)	*P* for trend	Mean (95% CI)	*P* for trend	Mean (95% CI)	*P* for trend	Mean (95% CI)	*P* for trend
BMI at the baseline^c^									
Normal (<25)	538	0.18 (0.04, 0.32)	0.004	0.18 (0.05, 0.32)	0.003	−0.03 (-0.18, 0.11)	0.79	−0.04 (−0.19, 0.10)	0.56
Overweight (25-30)	314	0.10 (−0.05, 0.26)		0.10 (−0.05, 0.26)		−0.01 (−0.17, 0.14)		−0.02 (−0.17, 0.14)	
Obese (>30)	145	−0.09 (−0.29, 0.10)		−0.09 (−0.29, 0.10)		−0.01 (−0.22, 0.19)		0.005 (−0.20, 0.21)	
Age group at the baseline^d^									
<50	163	0.01 (−0.17, 0.20)	0.75	−0.01 (−0.20, 0.18)	0.59	0.21 (0.01, 0.40)	0.05	0.22 (0.02, 0.41)	0.04
50-55	192	0.15 (−0.01, 0.31)		0.15 (−0.01, 0.31)		−0.01 (−0.18, 0.15)		−0.02 (−0.19, 0.14)	
55-60	222	0.25 (0.06, 0.45)		0.26 (0.06, 0.45)		−0.05 (−0.26, 0.15)		−0.07 (−0.28, 0.13)	
60-65	265	0.15 (−0.04, 0.35)		0.16 (−0.03, 0.35)		−0.08 (−0.28, 0.12)		−0.09 (−0.29, 0.11)	
>65	168	0.15 (−0.06, 0.37)		0.17 (−0.04, 0.38)		−0.14 (−0.36, 0.08)		−0.15 (−0.37, 0.07)	

a In the mtCN analysis, models were also adjusted for telomere length along with other variables indicated below.

b In the telomere analysis, models were also adjusted for mtCN along with other variables indicated below.

c General linear models are adjusted for age at blood draw, smoking, menopausal status, PMH use, and alcohol consumption.

d General linear models are adjusted for smoking, menopausal status, PMH use, and alcohol consumption.

Other demographic and lifestyle factors that are indicative of oxidative stress (including age, physical activity, alcohol consumption, and total calorie intake) were also examined in our study in relation to mtCN level, but no significant trend was detected (data not shown). Although we found an inverse correlation between mtCN and pack years of smoking in the Spearman test, no significant association was found for mtCN and smoking status (never, past, or current) (data not shown).

## DISCUSSION

Our study suggests that leukocyte mtCN is inversely associated with weight, waist size, BMI, and waist-hip ratio. Furthermore, weight gain was bi-directionally associated with decreased mtCN. Moreover, although mtCN was positively correlated with telomere length, mtCN appeared to be a stronger marker for obesity, while telomere length was more sensitive to age.

Oxidative stress is known to be a systemic problem in obese individuals. Cellular and molecular studies have revealed that obesity generates oxidative stress by mechanisms including hyperglycemia, elevated tissue-lipid levels, inadequate antioxidant defenses, chronic inflammation, excessive leukocyte infiltration and activation, endothelial ROS production, excessive renin-angiotensin system (RAS) hormone production, and hyperleptinemia. In every mechanism listed above, there is either elevated free-radical production or lowered antioxidant levels [28]. Oxidative stress may be directly caused by increased adiposity and fat distribution or consequent to behavioral changes associated with being obese. Population-based studies showed higher oxidative stress biomarkers in obese adults and children, especially among those with central or abdominal obesity. For instance, oxidation of LDL was found to be consistently and linearly associated with adiposity [28]; malondialdehyde (MDA) and F_2_-isoprostane concentrations, lipid peroxidation markers, were positively associated with BMI, body fat weight, visceral fat area, and total fat area [29, 30]; and plasma Thiobarbituric Acid Reactive Substances (TBARS) and urinary 8-epi-PGF_2_α were positively correlated with BMI and waist circumference [29].

The correlation between oxidative stress and leukocyte mtDNA copy number in healthy individuals was first evaluated in 2003, when positive correlations were observed between mtCN and various oxidative stress biomarkers in Chinese populations [31]. However, these findings could not be replicated in other ethnic groups [32]. In addition, *in vitro* experiments have shown that total mtDNA copy number in lymphocytes and whole blood did not increase in response to exogenous H_2_O_2_ treatments [33]. In epidemiologic studies, decreased mtCN levels are consistently found to be deleterious for many health outcomes, such as poorer self-rated health and cognitive ability, diminished physical strength, and higher prevalent frailty and all-cause mortality [5–7]. To date, the only association study between leukocyte mtCN and obesity was conducted among 94 young Korean subjects (mean age: 32.26±9.14 years), and found similar inverse associations between blood mtDNA copy number and BMI as well as waist circumference [34].

Our study revealed for the first time a bi-directional link between lower mtCN and weight gain. Although a causal relationship has not yet been established, there is an element common to both increased mtCN and efficient fatty acid metabolism: healthy, intact mitochondria. On one hand, mitochondria cannot remove or repair DNA damage caused to them by ROS. To compensate for the damage, healthy mitochondria increase their copy number in response to trans-acting factors encoded by nuclear DNA, possibly as a feedback mechanism to counterbalance the metabolic defects in mitochondria carrying mutated mtDNA and the resulting impaired respiratory system [8, 35]. On the other hand, mitochondria are the main sites for fatty acid β-oxidation (FAO) [2]. Loss of mitochondrial enzymes can cause obesity in mice [36]. Therefore, our finding that mtCN was bi-directionally and inversely associated with BMI might indicate a defect in mitochondrial function, by which mtCN can no longer be increased to cope with the stress caused by fatty acid metabolism.

Telomere length, another key marker reflecting the cumulative burden of oxidative stress and inflammation resulting from cellular and biological aging, was found to be a prime instigator of p53-mediated mitochondrial dysfunction in mouse models [18]. This evidence supports our finding that mtCN was positively associated with telomere length. However, our study did not replicate findings from previous studies that telomere length was inversely associated with anthropometric measurements, such as BMI and waist circumference [20–22]. To verify whether the inverse associations of mtCN with BMI identified in our study were due to differences in telomere length, we adjusted for it in our statistical models. However, the results remained significant, suggesting that mtCN might serve as a more sensitive biomarker for BMI than telomere length does. Regarding the effect of age on mtDNA alteration, several previous population-based studies demonstrated that mtDNA copy number was stable or positively correlated with age before an individual reached middle age (i.e., around 50). However, there may be a plateau stage after middle age, followed by a negative correlation [7, 31]. A similar pattern was observed in our data, though it was not statistically significant. In the meanwhile, our data also demonstrated a robust inverse correlation of telomere length with age even after adjustment for mtCN, as has been shown in previous studies [20–22]. Taken together, the evidence suggests that mtCN may be a stronger biomarker than telomere length with respect to obesity, while telomere length is more sensitive to age.

To the best of our knowledge, this is the first population-based study to examine the relationship between natural weight changes and leukocyte mtCN, and the inverse association between weight gain and mtCN level found in this study is in line with previous findings in previous telomere-length studies using similar age groups [37].

In conclusion, our results suggest that mtCN is inversely associated with weight, waist circumference, BMI, and weight-hip ratio, and that weight gain is bi-directionally associated with lower mtCN. In addition, although telomere length and mtCN are positively correlated, mtCN might serve as a more sensitive biomarker for BMI, while telomere length may be a stronger biomarker for age. The bi-directional temporal relationship between mitochondrial-mediated oxidative stress-defense mechanisms and weight change may suggest future strategies for weight management via reducing oxidative stress. For instance, in our study, lower mtCN is associated with weight gain and higher BMI, which may suggest a defective oxidative stress coping mechanism through mtCN. Reducing oxidative stress via lifestyle and dietary modification therefore could potentially protect mtDNA from cumulative damage, and thus maintain a functional fatty acid beta-oxidation. Future studies are warranted to confirm these findings and investigate the mechanisms.

## MATERIALS AND METHODS

### Study population and recruitment

Detailed descriptions of the NHS have been published elsewhere [23]. In brief, 121,700 registered nurses aged 30-50 years in 11 US states enrolled in the NHS in 1976. Participants completed a baseline questionnaire regarding risk factors for cancer and cardiovascular diseases and updated their information on lifestyle, medical history, and diet biennially. Between 1989 and 1990, blood samples were collected from 32,826 participants in the NHS. We included healthy controls from both lung- and skin-cancer case-control studies nested within the NHS in our study population (supplementary material). In the lung cancer study (n=321), one control per confirmed lung cancer case was selected, matching on age (±1 year), race, and smoking status and quantity at blood collection. In the skin cancer study (n=1,385), one control was matched to each confirmed skin cancer case by year of birth (±1 year) and self-reported race. The samples were limited to Caucasian women.

### Anthropometric measurements and other covariates

Exposures such as various anthropometric measurements as well as reproductive and lifestyle factors were included in the initial questionnaire and updated every two years in the NHS. Physical activity during the past year was assessed by questionnaire in 1988 and updated in 1992. The validity and reproducibility of these questions have been verified elsewhere [24]. Dietary intake in alternate cycles was assessed with a food frequency questionnaire (FFQ) and validated previously [25, 26]. Measurement of telomere length for the participants in this study has been published elsewhere [27].

BMI was calculated as weight in kilograms divided by the square of height in meters and was categorized as underweight (0 < BMI < 18.5), normal light (18.5<=BMI<23), normal heavy (23<=BMI<25), overweight (25<=BMI<27.5), pre-obese (27.5<=BMI<30), or obese (30<=BMI). Weight change since age 18 was defined as the difference between weight at blood collection (1989-1990) and the weight at age 18, categorized into four groups: weight loss (weight loss >2kg), weight maintenance (weight change −2kg to 2kg), weight gain 2-20kg, and weight gain >20kg. Weight change since baseline was defined as the difference between weight at blood collection and the weight recorded at the beginning of the NHS (1976), categorized into four groups: weight loss (weight loss >2kg), weight maintenance (weight change −2kg to 2kg), weight gain 2-10kg, and weight gain >10kg. Weight change since 1984 was defined as the difference between weight at blood collection and weight in 1984 (five years prior to blood collection), categorized into four groups: weight loss (weight loss >2kg), weight maintenance (weight change −2kg to 2kg), weight gain 2-5kg, and weight gain >5kg.

### MtDNA copy number ascertainment and validation

For quantitative PCR (qPCR)-based assay of relative mtDNA copy number, total DNA was extracted from buffy-coat fractions using the QIAmp (Qiagen, Chatsworth, CA) 96-spin blood protocol. DNA concentrations were determined via pico-green quantitation using a Molecular Devices 96-well spectrophotometer. Relative mtDNA copy number was assessed using a qPCR-based method in a high-throughput 384-well format with an Applied Biosystems 7900HT Real Time PCR system. DNA concentration was standardized to 5ng/μL. Genomic DNA (10ng per reaction) was added to a 384-well reaction plate and dried down. DNA was reconstituted in 10μL of multiplex ND2 (single-copy mitochondrial gene) and AluYb8 (nuclear repeat element) PCR reaction mixture. The 20 x multiplex reaction mixture consisted of 18μM each of the ND2-forward primer (5′-tgttggttatacccttcccgtacta-3′), ND2-reverse primer (5′-cctgcaaagatggtagagtagatga-3′), AluYb8-forward primer (5′-cttgcagtgagccgagatt -3′), and AluYb8-reverse primer (5′- gagacggagtctcgctctgtc -3′), and 5μM each of the ‘actgcagtccgcagtccggcct’ probe with VIC on the 5′ end and ‘MGBNFQ’ on the 3′ end, ‘ccctggcccaaccc’ with 6FAM on the 5′ end and ‘MGBNFQ’ on the 3′ end, plus 20 x multiplex Taqman genotyping mastermix (Taqman). The multiplex reaction thermal cycling profile was as follows: 95°C for 10 minutes, then 30 cycles of 95°C for 15 seconds and 60°C for 1 minute. Triplicate reactions of multiplex reactions were performed on each sample on different plates.

The average slope of the standard curves for both reactions was −3.5+/−0.3. The R^2^ coefficient of determination was 0.97 or higher for each reaction. The cycle threshold (Ct) value for each reaction represents the number of PCR cycles required to detect a signal over background fluorescence and is inversely proportional to the amount of DNA. The qPCR-based assay determined the mitochondrial ND2 gene copy number to genomic single-copy gene copy number (N/S) ratio, a value proportional to the average number of mtDNA copy number. The N/S ratio (−dCt) for each sample was calculated by subtracting the average AluYb8 Ct value from the average ND2 Ct value. The 10ng DNA standard curve point included on every 384-well plate was used as a calibrator to help adjust for inter-assay variability. The relative N/S ratio (−ddCt) was calculated by subtracting the N/S ratio of the calibrator DNA from the N/S ratio of each sample.

### Quality-control procedures

In addition to the samples, each 384-well plate contained a 6-point standard curve from 0.625ng to 20ng using pooled buffy coat-derived DNA. The purpose of the standard curve is to assess and compensate for inter-plate variation in PCR efficiency. We included 10% replicate quality control (QC) samples in the dataset to assess inter-plate and intra-plate variability of threshold cycle (Ct) values. The average inter-plate coefficients of variation for the ND2 and AluYb8 Ct values were 0.40% and 0.79% (respectively) among the quality-control samples. The average intra-plate coefficients of variation for the ND2 and AluYb8 Ct values were 0.50% and 0.90% (respectively) among the quality-control samples. The coefficent of variation for repeated samples was 7%, while within-person stability within one year showed a Spearman correlation of 0.4 and an ICC of 0.29.

### Statistical analysis

In this study, mtCN was assayed in various batches corresponding to each dataset. To minimize the impact of potential batch effect on leukocyte mtCN measurements across different datasets, we calculated z scores of log transformed leukocyte mtCN for each sample by standardizing the leukocyte mtCN in comparison with the mean within controls in each dataset. Levene's test for homogeneity and Welch's ANOVA test showed statistically homogeneous distributions of the z score from different datasets (*P*=1.00).

We examined age-adjusted correlations among different anthropometric measurements, smoking, physical activity, alcohol consumption, and mtCN z scores using Spearman partial rank correlation coefficients. To evaluate the potential dose-response relationships, adjusted least-squares mean mtCN z scores by categories of anthropometric measurements such as weight, height, waist and hip measurements, BMI (<25, 25<BMI<30 and >30), and waist-hip ratio were calculated using a generalized linear regression model controlling for age, menopausal status, postmenopausal hormone use, pack-years of smoking, physical activity (METs), and alcohol consumption (gm/day). We further examined the relationships between mtCN and weight changes. We also calculated adjusted least-squares mean mtCN z scores by categories of age at blood collection (<50, 50-55, 55-60, 60-65, and 65-71), smoking status (never, past, and current), and alcohol consumption (None, <5gm/day, 5-15gm/day, and 15+ gm/day). In addition, quartiles of telomere length z score and total calories were also calculated using generalized linear regression models, adjusting for menopausal status, postmenopausal hormone use, and lifestyle factors. We tested for linear trend across categories by including them as ordinal predictors in multivariate linear regression models. All analyses in this study were performed using SAS (Cary, NC), and statistical significance was set at *p*<0.05 (two-sided).
